# Lethal Experimental Tick-Borne Encephalitis Infection: Influence of Two Strains with Similar Virulence on the Immune Response

**DOI:** 10.3389/fmicb.2016.02172

**Published:** 2017-01-20

**Authors:** Anastasia S. Shevtsova, Oxana V. Motuzova, Vera M. Kuragina, Nelli K. Akhmatova, Larissa V. Gmyl, Yaroslava I. Kondrat'eva, Liubov I. Kozlovskaya, Yulia V. Rogova, Alexander G. Litov, Lidiya Iu. Romanova, Galina G. Karganova

**Affiliations:** ^1^Chumakov Institute of Poliomyelitis and Viral EncephalitidesMoscow, Russia; ^2^Federal State Budgetary Scientific Institution “I. Mechnikov Research Institute of Vaccines and Sera”Moscow, Russia

**Keywords:** tick-borne encephalitis virus, pathogenesis, dendritic cells, cytokines, interferon, immune response, flavivirus, non-sterile immunity

## Abstract

Tick-borne encephalitis virus (TBEV) is a tick-transmitted arbovirus that causes serious diseases in humans in Europe and Northern Asia. About 6000–10,000 cases are registered annually, and one-third of them lead to sequela with different degrees of severity. Two TBEV strains (Absettarov and EK-328) similar in virulence rate in laboratory mice were used to study pathogenesis and immune response upon lethal infection in mice. The strains differed in the dynamics of appearance of virus, IFNs and other cytokines in blood of mice, and ability to induce a cytokine storm in the terminal stages of disease and a non-sterile immunity. Moreover, the TBEV strains differed in characteristics of their interactions with DCs: level of reproduction in these cells, virus dose triggering IFN-α production, and impact on DCs' maturation. Infection of DCs with Absettarov strain led to IFN-α induction only at high multiplicity of infection (MOI), and an increased amount of the mature DCs with high adhesion activity and low-level of MHCII positive cells. While reproduction of the EK-328 strain in DCs was less efficient, a low dose of the virus induced IFN-α production and stimulated maturation of DCs with relatively low adhesive capacity, but with the high percentage of cells expressing MHCII molecules. Thus, the studied strains differed significantly in the impact on DCs' maturation and antigen presentation to CD4^+^ lymphocytes. Injection of low (10^3^ PFU) and high (10^6^ PFU) doses of both TBEV strains caused a lethal infection in mice. At the same time, the dose of the virus in the inoculum, regardless of the strain properties, affected the following virulence characteristics: the time of virus appearance in brain (day 4–5 vs. day 1 p.i.), time of IFN-α appearance in blood (10 h vs. 5 h p.i.), concentration of IFN-α in blood, and induction of IFN-α during infection of DCs. Therefore, virulent TBEV strains during lethal infection can interact differently with the host immune system, and the infectious dose has an impact on both: virus spread in the infected organism and immune response activation.

## Introduction

Tick-borne encephalitis (TBE) is one of the most serious arboviral human diseases in Europe and Northern Asia affecting the CNS. TBE is caused by a tick-transmitted mammalian *Flavivirus*, TBEV. Human infection usually occurs after a tick bite and results in cases with different outcomes, from asymptomatic infection to severe meningitis, meningoencephalitis, and encephalitis that can be fatal or can transform into a chronic disease.

TBE pathogenesis has been studied in detail on materials from patients with different forms of disease (Shapoval, 1961; Erman et al., 2001; Kaiser, 2008). Recently, activation of the immune system and brain inflammatory reaction in TBE patients has been estimated by measuring the level of the corresponding cytokines, enzymes, and other factors in blood and cerebrospinal fluid (Timofeev et al., 2002; Atrasheuskaya et al., 2003; Palus et al., 2015; Grygorczuk et al., 2016). Nevertheless, experiments in laboratory animals are required for evaluation of the pathogenic potential of the virus. Animal models allow reducing the influence of genetic diversity and immune status of test subjects and infectious doses of the virus and facilitate the ability to follow the initial stages of virus reproduction and activation of the immune responses.

There is a number of investigations that have studied TBEV pathogenesis in a laboratory mouse model using strains isolated from patients and from field-collected ticks, wild animals, and birds. The dynamics of virus distribution and reproduction in different organs, activation of the immune system, and virus interaction with cells of the immune system has been studied (Shubladze, 1939; Albrecht, 1968; Il'enko et al., 1974; Hayasaka et al., 2009; Ruzek et al., 2009; Tigabu et al., 2010; Tun et al., 2014).

Dendritic cells (DCs) play a special role in the initial stages of infection. Virus replication in DCs and virus manipulation of their maturation have been demonstrated for mosquito-borne flaviviruses: Dengue virus (Chase et al., 2011; Webster et al., 2016), Japanese encephalitis virus (JEV; Cao et al., 2011), West Nile virus (WNV; Rawle et al., 2015), and Yellow fever virus (YFV; Barba-Spaeth et al., 2005). There are some data for naturally attenuated tick-borne flavivirus Langat (LGTV; Robertson et al., 2014), but TBEV infection of DCs remains poorly characterized.

Many studies have been focused on comparison of highly virulent and attenuated TBEV variants in order to establish the features of the immune system activation in infected animals with different forms of disease, and to identify factors determining the pathogenetic characteristics of the virus. Authors used different approaches to evaluate the virulence for laboratory mice and to choose the appropriate infectious dose for compared viral strains. Usually, virus doses were based on plaque-forming units (PFU), sometimes on the amount of lethal doses (LD50) in the inoculum. The compared strains probably could vary significantly in the ratio of “virulent” virions capable of causing the disease in laboratory animals and “non-virulent” virions that could form plaques *in vitro* but were incapable of causing an acute infection *in vivo*, but still could influence the immune response. In light of this, the accuracy of the virus dose in the inocula comes under question. It is important to distinguish the differences related to the virus features, infectious doses, or composition of the virus population. The complexity of this problem determines the difficulty in creating a general picture of pathogenesis and immunogenesis during the experimental TBE. This information is important to understand the way of TBEV manipulations of mammalian immune response and can be crucial for antiviral drugs design.

In the present study we compared the pathogenesis and immune response after lethal infection caused by two TBEV strains similar in virulence rate in laboratory mice. It was shown that several characteristics were determined by the virus infectious dose. The virulent TBEV strains were found to vary greatly in their interactions with the host immune system, particularly with DCs.

## Materials and methods

### Cells

A pig embryo kidney (PEK) cell line was maintained in medium 199 (PIPVE, Russia) supplemented with 5% fetal bovine serum (FBS) (Gibco, Invitrogen, South America) at 37°C.

Murine DCs (bmDCs) were derived from bone marrow progenitor cells of BALB/c mice (female) after culturing in RPMI 1640 (PIPVE, Russia) supplemented with 10% heat-inactivated FBS, recombinant mouse IL-4, and GM-CSF (Invitrogen). Cytokines (20 ng/ml) were added on the days 1 and 3. After 7 days in culture, bmDCs were infected with viruses. DCs morphology was assessed by light microscopy.

### Viruses

TBEV strain EK-328 (GenBank ID DQ486861, Romanova et al., 2007) was isolated from *Ixodes persulcatus* in Estonia in 1972, and underwent eight passages in mouse brain with or without an additional passage in PEK cells.

TBEV strain Absettarov was isolated from the blood of a patient with TBE in the Leningrad region in 1951. This strain underwent about 20 passages in mouse brain with or without one passage in PEK cells. The full genome sequence of this strain (GenBank ID KU885457), except for the first 16 nucleotides (nt) of 5′-UTR and the last 60 nt of 3′-UTR, differed from the sequence of the strain called Absettarov (GenBank ID KJ000002) in 73 nt, of which 20 led to amino acid substitutions in all viral proteins except E and C, as well as to deletion of 272 nt in 3′-UTR.

Reference vaccine strain Sabin 1 of poliovirus type 1 (GenBank ID V01150) originated from NIBSC (UK).

Viruses were stored as aliquots of 10% infected mouse brain suspension or infected PEK cell culture supernate (CS) at −70°C.

### Flow-cytometry analysis

The fractions of DCs expressing different surface cell determinants (CDs) were measured by flow cytometry. At 72 h after TBEV infection, DCs were harvested, washed, and suspended in RPMI-1640 (PIPVE, Russia) with 1% BSA. Cell suspensions were labeled with the following anti-mouse antibodies (Abs): CD38-FITC (clone 90), CD83-FITC (clone Michel-17), CD86-PE (GL-1), CD80-PE (clone 16-10A1), CD11c-FITC (clone N418), MHCII (clone 14-4-4S) (BD Biosciences, USA), CD34-PE (clone MEC 14.7, Invitrogen, USA), and CD40-FITS (clone 3/23, BioLegend, USA) for 30 min at room temperature, washed, and fixed in 2% paraformaldehyde in PBS (Sigma-Aldrich). The percentage of cells expressing different CD molecules was analyzed using a FC-500 two-laser flow cytometer (Beckman-Coulter, USA). DCs incubated with 20 ng/ml TNF-α served as the positive control and bmDCs incubated with CS of uninfected PEK cells served as a negative control. The mean and standard deviation (s.d.) values for the experimental data were calculated. Mann-Whitney *U*-test was used to compare the percentage of infected cells vs. negative and positive controls.

### Immunofluorescent assay

DCs were stained with human serum obtained after immunization with inactivated TBE vaccine and labeled with FITC-conjugated anti-human IgG (Sigma-Aldrich). Stained cells, containing viral proteins, were counted using Nikon Eclipse Ti-U microscope equipped with fluorescent filters.

### Plaque assay and 50% plaque reduction neutralization test

The plaque assay procedure was described previously (Pripuzova et al., 2009). Briefly, PEK cells seeded in 6-well plates (Corning) were incubated with 10-fold dilutions of virus samples for 1 h. Then, each well was overlaid with 5 ml of 1% agar (Difco) on Earle's balanced salt solution (PIPVE, Russia) containing 7.5% FBS and 0.015% neutral red. After 6–14 days of incubation at 37°C plaques were counted and virus titers were calculated as the log_10_PFU per ml.

The 50% plaque reduction neutralization test (PRNT) was performed as described in Pripuzova et al. (2013): serial 2-fold dilutions of serum were prepared in 0.2 ml volumes. Virus diluted to contain approximately 30–40 PFUs was then added at a volume of 0.2 ml to each serum dilution, and mixtures were incubated at 37°C for 1 h. Then, the virus–serum mixtures were inoculated into wells with PEK cell monolayer in a volume of 0.4 ml, and the cells were incubated for 1 h for adsorption of non-neutralized virus. After incubation, each well was overlaid with agar (as described above) and incubated at 37°C for 5–6 days. Neutralizing antibody (Ab) titers were expressed as the highest serum dilution producing a 50% reduction of plaque count in comparison with the control, in which the test dose of the virus was incubated in the presence of serum from a non-infected mouse instead of examined serum.

### Virulence in mice

All experiments were carried out in 10–12-week-old female BALB/c mice (FSBI Scientific Center of Biomedical Technologies, Stolbovaya branch, Russia). The mice were maintained according to the international guidelines for animal husbandry, including the recommendations of Bankowski and Howard-Jones (1985) and the FELASA Working Group Report (1996–1997). The protocol was approved by the Chumakov IPVE ethics committee. TBEV strains were tested for either neurovirulence after intracerebral (IC) inoculation (30 μl) or for neuroinvasiveness after intraperitoneal (IP) inoculation (300 μl) or subcutaneous (SC) inoculation (100 μl; into the upper thigh). The mice were observed for 21 days after inoculation, clinical symptoms and deaths were recorded. LD50 was calculated according to the Kerber method (Lorenz and Bogel, 1973). The data were statistically processed using the Student test or Wilcoxon two-sample test.

### Detection of antiviral Abs in the mouse sera by ELISA

For antiviral Abs titration, ELISA was performed using the standard technique (Pripuzova et al., 2013) by the following scheme: 1st layer—non-inactivated virus antigen (Ag), 2nd layer—2-fold dilutions of the analyzed serum, 3rd layer—anti-mouse Abs (Anti-Mouse IgG IgA IgM (H+L) HRP-conjugated, Sigma-Aldrich).

Ag was prepared from concentrated CS of infected PEK cells using ultracentrifugation. The negative control Ag was prepared from CS of non-infected cells using the same method. Viral and negative control Ags were normalized by total protein content. The final Ab titer was calculated as the last serum dilution that gave an optical signal with the viral Ag 2-fold higher than the control Ag. All sera were tested in at least two replicates.

### ELISA for cytokine analysis

The concentrations of cytokines in mouse sera were detected by commercial ELISA kits (Genzyme and R&D Systems, Great Britain; Bio Source int., Belgium; PBL Biomedical Laboratories, USA) according to the manufacturer's instructions. The results were presented in pg/ml. Murine IFN-α in DCs supernate was measured by VeriKine Mouse IFN alpha ELISA Kit (Thermo Fisher Scientific Inc., USA). The data were statistically processed using the Student test.

### Real-time PCR

Viral RNA was extracted from 10% brain or spleen suspensions using TRI Reagent LS (Sigma Aldrich, USA) according to the manufacturer's instructions. Sabin type 1 poliovirus was used as an internal control and was added to the samples prior to RNA extraction. Reverse transcription was carried out with M-MLV reverse transcriptase (Promega, USA) according to the manufacturer's protocol with specific primers. Oligonucleotides for the Absettarov strain were used for reverse transcription and real-time PCR, as described previously (Schweiger and Cassinotti, 2003), and 3′-UTR was used as a target for real-time PCR. The oligonucleotides for the EK-328 strain and poliovirus were used for reverse transcription and real-time PCR, as described previously (Romanova et al., 2007), and NS5 was used as the target gene to perform real-time PCR for the EK-328 strain. Real-time PCR was carried out using a RealTime-PCR kit (Syntol, Russia). A standard curves were created using serial dilutions of TBEV RNA purified from CS of PEK cells by centrifugation in sucrose gradient as described previously (Gmyl et al., 2003).

## Results

### Virulence characteristics of studied TBEV strains in mice

For the present work, two TBEV strains, Absettarov and EK-328, with a high virulence rate in laboratory mice were selected (Kozlovskaya et al., 2010). The virus dose (LD50), expressed in PFU causing death of 50% of animals for both strains, slightly varied in different experiments even though the animals were from the same provider. In order to compare virulence of the investigated TBEV strains, data about virus titers in cell culture and in experiments in mice from several years were collected, and the variance between virulence rates in different experiments was evaluated. For each strain, data for virus obtained after reproduction in mouse brains (i.e., taken for the experiments in the form of brain suspension of infected mice) and after reproduction in cell culture (i.e., taken for the experiments in the form of CS from infected PEK cells) were analyzed.

For the Absettarov strain, the virus obtained after reproduction in mouse brain was more virulent than the one obtained after replication in PEK cells. IP injection of the virus from CS caused the death of 50% of animals with an infectious dose of 2.8 log_10_PFU, while the infectious dose for the virus obtained from the brain was 0.6 log_10_PFU (*P* < 0.05; Table 1, index c). The average life expectancies (ALE) of animals after IP infection with CS and brain viruses were similar; however, for animals SC infected with CS virus, the ALE was statistically higher (Table 1, index f).

**Table 1:** **Virulence rates of the Absettarov and EK-328 strains (represented as log_10_PFU) in BALB/c mice upon different routes of injection**.

**Strain**		**LD50 (log_10_PFU, M ± S)**			**Average life expectancy (days)**	
					**Inoculation of 10^3^ PFU**	
		**IC**	**IP**	**SC**	**IP**	**SC**
Absettarov	Brain	−1.2 ± 1.3 (3) a,b	0.6±0.4 (27) a, c	1.4±1.4 (8) b	10.0±0.2 [63] e	10.0±0.2 [29] f, g
	CS	n/a	2.8±0.2 (2) c	2.4±0.5 (2)	10.5±0.7 [4]	12.4±0.3 [10] f
EK-328	Brain	0.04 ± 0.3 (2)	0.7±0.6 (4) d	1.3±0.0 (6)	12.1±0.7 [34] e	13.4±0.7 [13] g
	CS	n/a	1.7±0.3 (5) d	1.6±0.2 (3)	10.1±0.7 [8]	14.0±1.9 [4]

*Mice were infected with the brain grown virus stocks*.

*M–geometric mean of titers in several experiments*.

*S–standard error*.

*()–number of experiments*.

*[]–number of animals taken into account to calculate average life expectancy*.

*n/a–not analyzed*.

*PFU–plaque forming unit*.

*CS–culture supernate*.

*IC–intracerebral injection*.

*IP – intraperitoneal injection*.

*SC – subcutaneous injection*.

*a–d–values indicated pairwise are significantly different according to Student's t-test (P < 0.05)*.

*e–g–values indicated pairwise are significantly different according to Wilcoxon two-sample test (P < 0.01)*.

For the EK-328 strain, a higher virulence rate of brain virus in comparison with CS virus was observed after IP inoculation (*P* < 0.05, Table 1, index d), but not after SC injection. A significant increase in ALE could not be established.

According to average calculations, a dependence of the virus virulence rate on the inoculation route for both strains could be observed. It decreased in series: IC to IP to SC injections. However, statistically significant differences in virus virulence rates were established only for IC inoculation of the Absettarov strain in comparison with IP inoculation (Table 1, index a) and SC inoculation (Table 1, index b). Noteworthy, the Absettarov strain population contained virions that could cause infection in mice after IC injection, but were unable to form a plaque in PEK cells (1LD50 corresponded with 0.1 PFU).

The average virulence rates were similar for both strains, but ALE for the brain variant of the EK-328 strain was significantly higher (*P* < 0.01) than the one for the Absettarov strain upon IP and SC inoculation routes (Table 1, indexes e and g).

### Characteristics of the mice infection

First, brain grown virus stocks of both investigated viruses were compared upon IP injection, when the two strains demonstrated the most similar characteristics. The infection parameters were analyzed after IP inoculation of 10^3^ PFU of both viruses. Daily three of infected mice were autopsied without perfusion, but after total bleeding, and samples of brain, spleen, and inguinal and popliteal lymph nodes were collected. Titers of infectious virus in the samples were measured by plaque assay in PEK cells. In previous experiments, we have found that the titers of the infectious virus in blood clots are similar or higher than in sera (Pripuzova et al., 2009). Therefore, to estimate the viremia in this experiment, we utilized 10% blood clot suspensions. Serum pools from three animals were used to determine the levels of selected cytokines and Abs.

**Figure 1:**
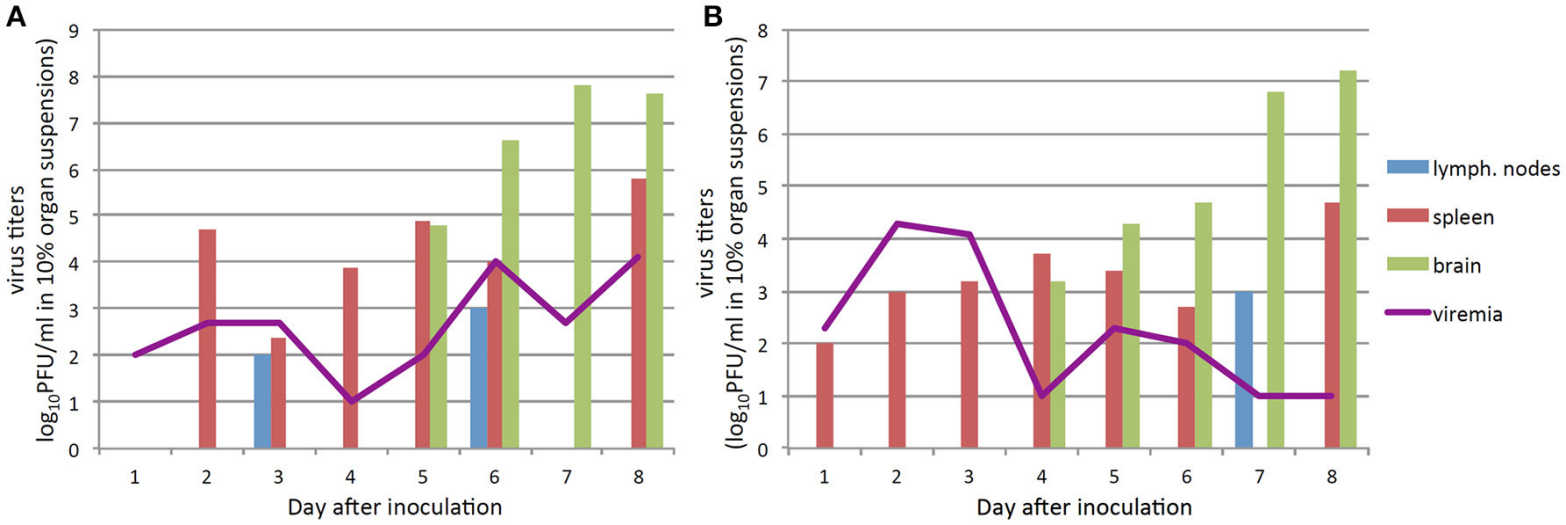
**The infectious virus detection in blood, brain, spleen, and lymph nodes of infected mice (pool from three animals; inoculated virus in the form of brain suspension) at different time points post infection:** the animals IP infected with 10^3^ PFU of TBEV Absettarov strain **(A)**, with 10^3^ PFU the EK-328 strain **(B)**. Virus yields in 10% tissue suspensions were determined by plaque assay in PEK cells.

In this experiment, symptoms of the disease developed on days 6–7 after IP infection with the Absettarov strain, and by day 9, all animals died. We observed two waves of viremia (Figure 1A). Infectious virus titers in the peripheral lymph nodes correlated with the highest virus titers in blood of infected animals and were detected on days 3 and 6 post-infection (p.i.) (Figure 1A). The virus appeared on day 2 p.i. in spleen. In brain, virus appeared on day 5 p.i., which coincided with the second peak of viremia.

After IP infection of 10^3^ PFU of the EK-328 strain, acute infection with two-wave viremia was also observed, but the first wave was higher (Figure 1B). Infectious virus was detected in the peripheral lymph nodes on day 7 p.i., but in spleen the virus was observed from the first day (Figure 1B). Infectious virus appeared in brain of mice infected with the EK-328 strain 1 day earlier than in Absettarov-infected animals. The animals got sick on days 7–9 p.i. and died within 2–3 days after the disease onset.

Thus, the virus was detected in brain 4–5 days p.i., and two-wave viremia was demonstrated for both TBEV strains; however, the viruses differed in the relation of the peaks.

### The selected cytokines dynamics in mouse serum

Pooled sera from three mice were used to evaluate immune response activation after IP inoculation of brain grown stocks of the TBEV strains in a dose of 10^3^ PFU. The virus distribution in organs of these animals was described above. The levels of selected cytokines were measured in serum using ELISA and compared with the cytokine levels in the sera of mice IP inoculated with brain suspension from a non-infected mouse in the corresponding dilution (Table 2a).

**Table 2a:** **The dynamics of the selected cytokines appearance in sera of infected mice**.

**Cytokines (pg/ml)**	**Day of infection**													
	**1**		**2**		**3**		**4**		**5**		**6**		**7**	
	**Abset**	**EK**	**Abset**	**EK**	**Abset**	**EK**	**Abset**	**EK**	**Abset**	**EK**	**Abset**	**EK**	**Abset**	**EK**
TNF-α	–	–	–	–	–	–	–	–	–	107	–	–	315 ± 43	n/a
IL-10	101 ± 34	–	9 ± 7	–	14 ± 7	–	–	–	–	–	–	–	45 ± 14^*^	33 ± 1^*^
IFN-γ	–	–	–	–	–	30 ± 2	27 ± 5	49 ± 11	39 ± 10	–	–	–	284 ± 36	n/a
IL-12	–	–	328 ± 17	–	–	–	–	292 ± 31	n/a	n/a	n/a	n/a	n/a	n/a
IL-6	–	–	–	–	–	74 ± 6	69 ± 9	–	–	–	–	–	640 ± 85	113 ± 9
IL-2	–	–	–	–	–	–	–	–	–	–	–	–	–	n/a

*Animals were IP infected with 10^3^ PFU of the brain grown stocks of the Absettarov and EK-328 strains*.

*n/a–not analyzed*.

“*–”– concentration of cytokine lower than in control serum, which was obtained from animals after injection of brain suspension from non-infected animals with subsequent dilution (pg/ml): TNF-α, 23 ± 5; IL-10, 5 ± 2; IFN-γ, 25 ± 2; IL-12, 131 ± 56; IL-6, 16 ± 1; IL-2, 10 ± 2*.

* *Indicates the data for sick animals; for mice without clinical manifestation, the concentration of the interleukin on the indicated day was lower than in the control*.

Starting from the first day of severe infection caused by 10^3^ PFU Absettarov strain, no TNF-α or IL-2 were detected in sera of infected mice (Table 2a). IL-10 production increased on the first day p.i. and decreased by day 4. IL-12 appeared on day 2, IFN-γ on days 4–5, and IL-6 on day 4. On day 7 p.i., when the first symptoms of the disease appeared, an increase in IFN-γ, IL-6, IL-10, and TNF-α was found, which could be considered as a cytokine storm.

In the infection caused by 10^3^ PFU EK-328 strain, IL-10 was not detected over the first 6 days p.i. (Table 2a). IFN-γ and IL-6 were detected by 1 day earlier than in Absettarov-infected mice. We also observed an increase in IL-12, IFN-γ, and TNF-α concentration on days 4–5 p.i. On day 7, an increase in IL-10 and IL-6 production was observed, but it was less pronounced than in Absettarov-infected mice.

The obtained data indicated that during severe form of experimental TBE infection, days 3–4 p.i., when INF-γ and IL-6 appeared in the bloodstream, were a critical moment for the development of the specific immune response. IFN-γ restores the balance between Th1 and Th2 lymphocytes and suppresses IL-10 production (Hu et al., 2006; Cope et al., 2011), whereas IL-6 increases proliferation and differentiation of B-lymphocytes, which results in specific Abs production.

In additional experiment four mice were infected with high (10^6^ PFU) doses of both strains. On day 6 p.i., IFN-γ and IL-6 were assayed in individual serum samples from the sick animals. As shown in Table 2b, sera of all infected animals contained high concentrations of both cytokines. The IFN-γ/IL-6 ratios were 0.4 and 0.5 for the Absettarov and EK-328 strains, respectively, vs. 1.5 in control samples. Thus, Th2 cells might play the leading roles in Th1/Th2 immune balance in pathogenesis of TBE caused by either of these two strains.

**Table 2b:** **Concentration of cytokines in sera of infected mice (6 d p.i.)**.

**Cytokine (pg/ml)**	**Absettarov**	**EK-328**	**Control**
IFN-γ	38 ± 7	48 ± 3	12 ± 3
IL-6	108 ± 68	96 ± 35	8 ± 1
INF-γ/IL-6	0.35	0.5	1.5

*Animals were IP infected with 10^6^ PFU of the brain grown stocks of the Absettarov and EK-328 strains*.

Moreover, we could observe a cytokine storm at the terminal stage of infection. These highly virulent TBEV strains differed in the dynamics of the appearance of cytokines in the serum of infected mice and a cytokine storm.

### The dynamics of the appearance of antiviral Abs in mouse serum after IP inoculation

Sera from infected mice, obtained during the experiment described above, were assayed in ELISA for the presence of antiviral Abs. As shown in Table 3, Abs appeared on day 4 p.i. The level of antiviral Abs and their dynamics were similar for both studied TBEV strains.

**Table 3:** **Titers of antiviral Abs in the sera of infected animals in ELISA**.

**Strain**	**Day of infection**						
	**1**	**2**	**3**	**4**	**5**	**6**	**7**
Absettarov	0	0	0	1:160	1:160	1:320	1:320
EK-328	0	0	0	1:160	1:160	1:160	1:320

*Mice were IP infected with 10^3^ PFU of the brain grown stocks of the Absettarov and EK-328 strains*.

*Sera samples were polled from three mice*.

The same sera pools from infected animals taken on day 6 p.i. (Figure 1) were analyzed in the PRNT. The titers of virus-neutralizing Abs in mouse blood were similar for both viruses (Table 4). In addition to neutralizing Abs, 2.1 log_10_PFU/ml of infectious virus was detected in the sera of Absettarov-infected mice. Titers of the infectious virus were very low in blood of EK-328-infected mice. Therefore, the non-sterile immunity phenomenon was observed on the second wave of viremia, when neutralizing Abs and infectious virions co-circulate in bloodstream. Moreover, the studied strains differed in this property.

**Table 4:** **Geometric mean titers (GMTs, log_10_) of neutralizing Abs in mice sera on the 6th day after TBEV inoculation**.

**Sera used in neutralization test**	**Viruses used in neutralization test**	
	**Original virus from inoculate**	**Virus obtained from brain of infected mouse**
**ABSETTAROV STRAIN**		
Serum of mouse infected with strain Absettarov	2.2 ± 0.1	2.2 ± 0.2
**EK-328 STRAIN**		
Serum of mouse infected with strain EK-328	2.4 ± 0.1	1.6 ± 0.2

*Mice were IP infected with 10^3^ PFU of the brain grown stocks of the EK-328 and Absettarov strains*.

*Serum and brain were taken from infected animals on the 6 d p.i*.

In additional experiments, we examined the ability of the serum Abs to neutralize the virus used for inoculation and the virus from mouse brains taken during infection. We investigated the sera of mice taken on day 6 after IP injection of 10^3^ PFU of the Absettarov and EK-328 strains in the PRNT. Brain samples were collected simultaneously with the sera from the same animals. For EK-328-infected animals, the titer of Abs neutralizing the virus used for inoculation was higher (*P* < 0.05) than the ones neutralizing the virus from the brain of this mouse (Table 4), while Abs in the sera of mice infected with the Absettarov strain efficiently neutralized both viruses.

### The detailed characteristics of the first stages of infection

The main events associated with innate immunity activation and, consequently, with specificity of infectious processes occur during the first stages of infection. In further investigation, we used CS from infected PEK cells to prevent the potential impact of even trace amount of brain suspension in the inoculum on the results. The control group comprised mice injected with CS from non-infected PEK cells.

We evaluated the first stages of virus infection after IP inoculation of 10^3^ and 10^6^ PFU of the cell culture grown stocks of the investigated strains. At different time points, samples of blood, brain, and spleen were taken from 3 to 5 mice infected with each virus. The presence of viral RNA was assayed by real-time PCR in 10% spleen and brain suspension separately for each animal. The 10% blood clot suspensions were used for virus titration by plaque assay. The level of cytokines was measured in the sera of infected mice by ELISA.

The dynamics of virus appearance in blood during the first stage of infection was similar for both viruses. The infectious virus was detected 5 h p.i. for both infectious doses (Table 5a); however, the virus titers were higher in blood of mice infected with 10^6^ PFU of viruses. Assuming that TBEV reproduction cycle is longer than 12 h, we could estimate that the infectious virus detected 5 h p.i. is the inoculated one.

Table 5**The dynamics of virus appearance in blood, brain and spleen of mice during early stages of TBEV infection**.

**Table:** 

**a. Viremia after IP inoculation of the Absettarov and EK-328 strains (plaque assay)**.							
**Strain**	**Dose in inoculum (PFU)**	**5 h**		**10 h**		**28 h**	
		Detection^*^N = 3	GMT^**^ (log_10_PFU/ml)	Detection^*^N = 3	GMT^**^ (log_10_PFU/ml)	Detection^*^N = 5	GMT^**^ (log_10_PFU/ml)
EK-328	10^3^	1	1.4	0	–	3	1.9 ± 0.3
	10^6^	3	3.0 ± 0.6	3	1.9 ± 0.3	5	4.0 ± 0.2
Absettarov	10^3^	3	1.6 ± 0.1	0	–	3	1.9 ± 0.0
	10^6^	3	3.7 ± 0.1	3	2.8 ± 0.2	5	3.8 ± 0.1

**Table:** 

**b. Geometric mean of viral titers and number of viral RNA copies in the brains of animals IP infected with the Absettarov and EK-328 strains (28 h p.i.)**.					
**Strain**	**Dose in inoculum (PFU)**	**Plaque assay**		**RT-PCR**	
		Detection^*^N = 3	GMT^**^ (log_10_PFU/ml)	Detection^*^N = 3	GMT^**^ (log_10_RNAs/ml)
EK-328	10^3^	0	–	0	–
	10^6^	2	0.8 ± 0.3	2	4.9 ± 0.2
Absettarov	10^3^	0	–	0	–
	10^6^	3	0.8 ± 0.1	0	–

**Table:** 

**c. Viral RNA in spleens of infected mice (RT-PCR)**.							
**Strain**	**Dose in inoculum (PFU)**	**5 h**		**10 h**		**28 h**	
		Detection^*^N = 3	GMT^**^ (log_10_ RNAs/ml)	Detection^*^N = 3	GMT^**^ (log_10_ RNAs/ml)	Detection^*^N = 3	GMT^**^ (log_10_ RNAs/ml)
EK-328	10^3^	0	–	0	–	0	–
	10^6^	3	4.0 ± 0.8	1	4.7	3	4.9 ± 0.2
Absettarov	10^3^	0	–	0	–	0	–
	10^6^	3	5.4 ± 0.6	3	6.4 ± 0.2	3	4.2 ± 0.5

*Mice were infected with cell culture grown virus stocks*.

*GMT–geometric mean titer*.

*N–total number of animals taken for experiment*.

* *–the number of animals in which blood (brain, spleen) the virus was detected*.

** *–in 10% tissue (brain, spleen) suspension*.

“*–” not detected*.

*Panel b–Real Time PCR sensitivity 1000 RNAs/sample*.

*Panel c–Real Time PCR sensitivity 3000 RNAs/sample*.

Twenty-eight hours p.i. of the high dose (10^6^ PFU), real-time PCR and plaque assay revealed infectious virus in brains of two out of three EK-328-infected mice and three out of three Absettarov-infected mice. The infectious virus titers were very low and similar for both investigated viruses. After inoculation of the low virus dose (10^3^ PFU), the infectious virus was not detected in the mouse brain for either studied strains (Table 5b). The level of viral RNA in spleen after the low dose infection was below the test sensitivity threshold for both studied viruses (Table 5c).

Thus, for both TBEV strains the virus titers in spleen and brain varied with the infectious dose. After inoculation of the high dose, the infectious virus appeared in spleen at 5 h p.i. and brain of infected animal at 28 h p.i.

### Activation of the innate immune response on the first stages of infection after IP inoculation of the TBEV strains

The levels of TNF-α, IL-10, and IL-12 in blood serum of three animals infected by IP inoculation of the cell culture grown stocks of the Absettarov or EK-328 strains were measured by ELISA (Table 6). We detected IL-10 and TNF-α in mouse sera 10 h after Absettarov-strain infection. Later, concentration of TNF-α decreased to the control level. In case of EK-328-infected mice, TNF-α and IL-10 appeared in sera only in 18 h p.i, as the increase in IL-12 production was detected in 10 h p.i.

**Table 6:** **The dynamics of cytokines appearance in mouse sera during the early stages of TBEV infection**.

**Strain**	**TNF-α (pg/ml)**				**IL-10 (pg/ml)**			**IL-12 (pg/ml)**	
	**5 h**	**10 h**	**18 h**	**24 h**	**10 h**	**18 h**	**24 h**	**10 h**	**24 h**
Absettarov	–	107 ± 19	–	–	301 ± 10	20 ± 0	–	–	–
EK-328	–	–	70 ± 9	–	–	75 ± 5	–	352 ± 42	–
Control	23 ± 3				5 ± 2			131 ± 56	

*Mice were IP infected with 10^3^ PFU of the cell culture grown stocks of the Absettarov and EK-328 strains*.

“*–”– concentration of cytokines lower or equal to the value in control sera obtained from animals after injection of CS of non-infected PEK cells*.

TNF-α, a pro-inflammatory cytokine, indicates macrophage and neutrophil activation. IL-10 is induced by TNF-α and, in turn, down-regulates TNF-α synthesis (Platzer et al., 1995; Brennan et al., 2008). Induction of IL-12 during the initial innate immune responses elicits activation of natural killers, promotes IFN-γ production, and increases CD4^+^ T-cell differentiation (Tominaga et al., 2000). Activation of an adaptive immune response that could be followed by the appearance of IL-6 and IFN-γ in sera was detected by 1 day earlier in EK-328-infected mice than in Absettarov-infected ones (Table 2a).

Thus, even though the TBEV strains did not differ by the time of virus appearance in brain, spleen, and blood of infected animals during the first day of infection, they showed significant differences in the dynamics of pro-inflammatory cytokines appearance in blood serum.

### The dynamics of the interferon synthesis in mouse serum in the first stages of TBEV infection

The rate of interferon synthesis during the first stages of infection was assessed using ELISA. The concentration of cytokines in blood sera were averaged over four animals.

The concentration of IFN-α in serum of infected mice was dose-dependent for both viruses (Table 7a). After the low dose of infection (10^3^ PFU), we failed to detect this cytokine. After infection with EK-328 strain in the high dose (10^6^ PFU), IFN-α was detected at 10 h p.i. After injection of 10^6^ PFU of the Absettarov strain, IFN-α appeared at 5 h p.i. followed by a 1.5-fold increase at 10 h p.i. The Absettarov strain induced higher titers of IFN-α production at all time-points.

Table 7**The dynamics of interferons appearance in sera of TBEV infected mice**.

**Table:** 

**a. Concentration of IFN-α in the infected mice sera**.				
**Strain**	**Dose in inoculum (PFU)**	**IFN-α (pg/ml)**		
		**5 h**	**10 h**	**28 h**
EK-328	10^3^	–	–	–
	10^6^	–	584 ± 10	194 ± 5
Absettarov	10^3^	–	–	–
	10^6^	424 ± 90	711 ± 13	488 ± 10
Control		38 ± 8	11 ± 4	57 ± 14

**Table:** 

**b. Concentration of IFN-γ in the infected mice sera**.				
**Strain**	**Dose in inoculum**	**IFN- γ (pg/ml)**		
		**5 h**	**10 h**	**28 h**
EK-328	10^3^	–	14 ± 4	–
	10^6^	–	45 ± 12	–
Absettarov	10^3^	–	–	19 ± 7
	10^6^	–	63 ± 3	–
Control		–	–	5 ± 0

*Mice were infected with cell culture grown virus stocks*.

“*–”– concentration of cytokines lower or equal to the value in control sera obtained from animals after injection of non-infected cells CS*.

The EK-328 and Absettarov strains induced production of IFN-γ at 10 h after high-dose infection and reduction of IFN-γ synthesis at 28 h p.i. (Table 7b). The concentration of IFN-γ was lower in the sera of EK-328-infected mice than in the sera of Absettarov-infected ones. The low dose of the Absettarov strain induced the synthesis of IFN-γ only at 28 h p.i. and low dose of EK-328 virus at 10 h p.i. This correlated with the detection of IL-12 in the sera of animals infected with the low dose of EK-328. Therefore, the level of IFN-γ production after low-dose infection may depend on virus features.

### Interaction of TBEV strains with dendritic cells (DCs)

During the first stages of viral infection, DCs play an important role in the initiation of the innate immune response (Ag presentation and production of IFN-α). We compared reproduction dynamics of the EK-328 and Absettarov strains and evaluated their effects on DCs' maturation and IFN-α synthesis. Murine DCs obtained by adding IL-4 and GM-CSF to precursor cells from the mouse bone marrow were used. On day 7 of stimulation, DCs were infected with different doses of CS of PEK cells infected with studied strains. The populations of DCs were evaluated by the percentage of CD-marker positive cells using flow cytometry. DCs incubated with CS from non-infected PEK cells were used as a negative control. DCs incubated with TNF-α were used as a positive control.

**Figure 2:**
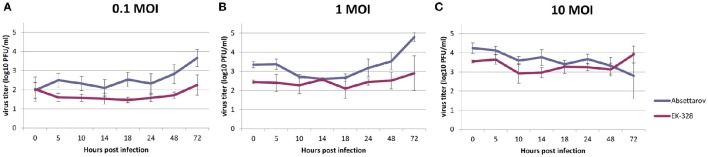
**The dynamics of reproduction of the Absettarov and EK-328 strains in murine DCs (mean for four experiments) at different MOI** [**(A)** 0.1, **(B)** 1, and **(C)** 10 PFU/cell]. Virus yields in culture supernatant were determined at different time points post infection by plaque assay in PEK cells.

In order to estimate the ability of the viruses to reproduce in DCs, DCs were infected with high (10 PFU/cell), medium (1 PFU/cell), and low (0.1 PFU/cell) MOI of TBEV strains. Both strains showed the level of reproduction in DCs of no more 5 log_10_ PFU/ml (data from four experiments). The absolute values of virus titers changed from one experiment to another. Nevertheless, all experiments had a similar profile. Figure 2 represents the dynamics of infectious virus accumulation in the CS of DCs (mean data from four experiments).

Although, cells were washed from non-attached viruses after adsorption, at the zero time point quite high titers of the virus were detected that remained attached to the cell surface and did not enter the cell. In all experiments, infection with high MOI of both TBEV strains led to a slight variation in virus titers starting from day 2 p.i. However, medium and low MOI provoked an increase in virus titers in the CS. Titers in CS of Absettarov-infected DCs were higher than the ones infected with the EK-328 strain at low and medium MOI at 72 h p.i. in all experiments (statistical significance is confirmed by the sign test). The number of infected DCs was estimated by immunofluorescent assay at 24 and 48 h p.i. using human polyclonal immune serum. At 24 h p.i., the number of infected cell was negligible even at high MOI. At 48 h p.i., the cells infected with high MOI of the Absettarov and EK-328 strains constituted 0.5 and 1.7%, respectively.

**Figure 3:**
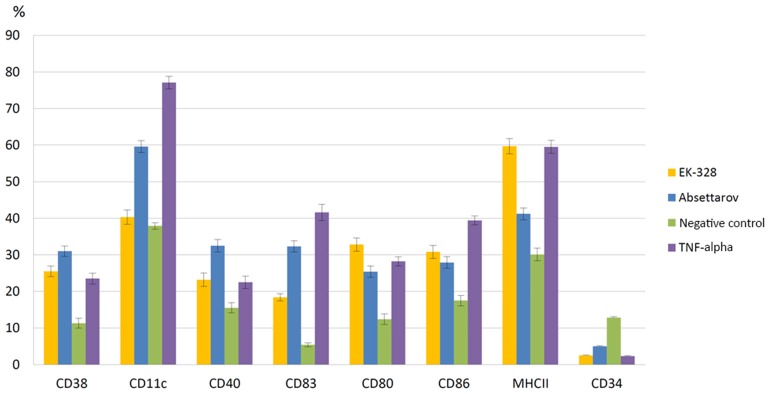
**Percentage of DCs with different CD markers at 72 h after infection with the Absettarov and EK-328 strains**. DCs were infected with viruses at MOI 10 PFU/cell. The fractions of DCs expressing different surface CDs were measured by flow cytometry.

Phenotyping showed that culturing of mouse bone marrow precursor cells in the presence of IL-4 and GM-CSF (negative control) increased percentage of DCs carrying immature cells marker CD34, which indicated the functional immaturity of non-infected DCs (Figure 3). Addition of TNF-α, a classical inducer of maturation, to the cell culture (positive control) led to a significant (*p* < 0.05) decrease of number of CD34+ DCs in comparison with the negative control and an increase (*p* < 0.05) in the content of cells expressing adhesion markers CD38 and CD11c; co-stimulatory molecules CD80, CD86, and CD40; molecules of Ag presentation MHCII; and terminal differentiation CD83 (Figure 3).

Infection of immature DCs with the EK-328 strain was followed by a statistically significant increase in the content of cells carrying adhesion marker CD38; co-stimulatory molecules CD80, CD86, and CD40; terminal differentiation CD83; and molecules of Ag presentation MHCII in population compared to the negative control. At the same time, the content of cells with CD11c molecule remained at the negative control level, meaning that cells that corresponded to mature DCs by their phenotypical characteristics had low adhesive capacity (Figure 3).

Infection of immature DCs with the Absettarov strain led to even more significant increase in the percentage of CD38 (*p* < 0.05), CD40 (*p* < 0.05), CD83 (*p* < 0.05), and CD11c (*p* < 0.05) positive cells in comparison with the negative control, but percentage of cells expressing MHCII molecule was considerably lower that after infection with EK-328 strain. Moreover, the Absettarov strain induced differentiation of the MHCII-positive cells (compared to the control, *p* < 0.05). A peculiarity of the Absettarov strain in comparison with the EK-328 strain was its ability to stimulate expression of CD11c molecules on DC's surface (Figure 3).

In three experiments, the ability of the viruses to induce IFN-α after DCs' infection was estimated. The obtained data showed significant variability in both: absolute levels of IFN-α and number of samples with detectable levels of IFN-α (Table 8). However, in DCs infected with the high MOI of the Absettarov strain (10 PFU/cell) and the low MOI of the EK-328 strain (0.1 PFU/cell), IFN-α was detected within 5 h p.i. in all experiments. In none of the experiments, IFN-α was detected within 5 h p.i. at the low and medium MOI of the Absettarov strain, and within 10 h p.i. at the high MOI of both strains.

**Table 8:** **IFN-α detection in supernates of infected DCs within 5 and 10 h after TBEV infection at different MOI**.

**Strain**	**MOI (PFU/cell)**	**5 h p.i. (Mean, pg/ml)**	**10 h p.i. (Mean, pg/ml)**
Absettarov	0.1	0/3^*^	1/3 (13)
	1	0/3	1/3 (47.7)
	10	3/3 (20.4 ± 7.1)	0/3
EK-328	0.1	3/3 (18.6 ± 6.5)	2/3 (15.6 ± 0.6)
	1	0/3	1/3 (15.4)
	10	1/3 (17.9)	0/3

* *Number of IFN-α ELISA positive samples (in numerator), number of experiments (in enumerator)*.

The obtained data suggest that IFN-α induction during TBEV infection of DCs occurs at the early stages of infection, and the inducing dose depends on the TBEV strain.

## Discussion

A clarification of the factors determining the outcome of the human contact with TBEV is a key for understanding of the pathogenesis of infection, identification of virulence determinants, and even evaluation of the epidemiological situation in the natural foci (natural focus of an infectious disease is a minimal territory of one or several landscapes, in which the pathogen circulation maintains without importations for an indefinitely long period of time, Kucheruk and Rosický, 1983). A lot of factors can define the severity of the disease: genetic predisposition and immune status of the mammalian host during the tick bite, and some features of virus infection, including infectious dose, properties of the infectious virus, time of the tick feeding, location of the tick bite, composition and amount of tick saliva, additional tick-borne pathogens, etc. Nevertheless, the role of virus properties in the disease severity remains unclear. Model experiments in inbred laboratory animals allow reducing the influence of several from above-mentioned factors. However, the first stage of a study should solve two problems: elucidate and divide the effects, determined by the infectious dose and virus features; and estimate the homogeneity of virus variants by interaction with the host immune system.

In comparative experiments it can be difficult to equilibrate different strains by infectious dose, because the virus populations can vary in the percentage of the virions able to cause an acute infection in mice. To avoid these difficulties two TBEV strains were chosen for the present work: the Absettarov European subtype strain and the EK-328 Siberian subtype strain, because they demonstrated similar virulence rate in laboratory mice (Kozlovskaya et al., 2010), i.e., PFU count sufficient to cause death of 50% of animals.

The virus inoculation route is very important in the study of the dynamics of the infectious process as well as for characterization of the immune response activation. As it has been demonstrated previously, in a short time after SC injection, the virus can be found in fibroblasts and in the hypoderm near the site of inoculation (Shi-Gie and Pogodina, 1964; Albrecht, 1968; Labuda et al., 1996). Virus particles captured by Langerhans cells are transported to the lymph nodes (Labuda et al., 1996; Banchereau and Steinman, 1998). After IP inoculation, the virus can appear in the blood bypassing reproduction at the site of injection. In this case, the first stages of the virus reproduction mainly occur in the blood cells. Both virus administration routes only partially simulate the process of natural infection after tick bite. For our experiments, we chose IP injection.

Our data demonstrate that the characteristics of the infection and activation of the immune response after IP inoculation significantly depend on the virus dose in the inoculum. For both TBEV strains, IP injection of low (10^3^ PFU) and high (10^6^ PFU) doses caused lethal infection. At the same time, the dose of the virus in the inoculum, regardless of the strain properties, affected the following characteristics of the infection: the time of virus appearance in brain (day 4–5 vs. day 1 p.i.; Table 5b, Figures 1A,B), time of IFN-α and IFN-γ appearance in blood (10 vs. 5 h p.i.; Table 7a,b), concentration of IFN-α in blood (Table 7a), and induction of IFN-α during infection of DCs (Table 8).

Previous studies have shown virus appearance in the CNS of mice 24 h after SC injection of a high dose of highly virulent (Shubladze, 1939) and relatively low virulent (Hayasaka et al., 2009) TBEV strains. In our work, the IP injection of two highly virulent strains in the low dose led to a virus appearance in the CNS on day 4–5 p.i., concurrently with the Th2 type specific immunity activation and synthesis of antiviral Abs. After virus injection in the high dose, detectable amounts of the virus appeared in the CNS on the 1st day of infection, i.e., before activation of specific immunity. Therefore, the infectious dose could determine the rate of virus penetration into the CNS, and, apparently, the severity and character of CNS damage caused by cytotoxicity of immunocompetent cells.

The IFN-α system appears to play a key role in activation of the innate immunity. It affects activation of immunocompetent cells and induction of other pro-inflammatory cytokines (Haller et al., 2006). In our experiments the high infectious dose (10^6^ PFU) induced IFN-α synthesis within 5–10 h p.i. At the same time, the relatively low infectious dose (10^3^ PFU) could cause death of all animals, but was unable to activate the production of type I IFN to detectable levels.

Viral infection activates IFN production through TLR3 in almost all types of cells (Takeda and Akira, 2005). TLR3 recognize viral dsRNA in the extracellular environment or in the cytoplasm. Overby et al. (2010) showed that TBEV infection delayed activation of IRF-3 and induction of IFN. They hypothesized that the virus rearranged the internal cell membranes to provide a compartment for its dsRNA that became inaccessible for recognition. Alternatively, IFN induction during viral infection is possible through TLR7 and TLR9 in DCs. TLR7 activates in the presence of ssRNA in the cell endosomes or after DC' contact with a virus-infected cell (Takeda and Akira, 2005; Webster et al., 2016). It is known that plasmacytoid DCs produce 95% of IFN (Fitzgerald-Bocarsly, 2002). We could assume that in our case, IFN synthesis was activated via TLR-7 in plasmacytoid DCs. To verify this hypothesis, we used murine DCs obtained after stimulation of bone marrow precursors and showed that these cells could synthesize IFN-α in a dose-dependent manner at the very first stages of the infection.

Infection with both studied TBEV strains in the low dose was not accompanied by IFN type I accumulation in mouse blood; however, infection of DCs with the low dose of the EK-328 strain led to production of IFN-α within 5 h p.i. This could indicate that subpopulations of DCs undergo activation differently *in vivo* and *in vitro* or that the probability of DC-virus contact dramatically decreased at the low infectious dose. The latter is highly possible, because the amount of DCs in the blood is less than 1% (Fitzgerald-Bocarsly, 2002). Thus, the type of cells interacting with the virus during the first stage of infection, and the coordination of the immune response can depend on the virus infectious dose.

The characterization of immune response activation after injection of different viral doses requires further investigation. At the same time, our data suggest that an infectious dose can significantly affect the character of the immune response activation. It is obvious, when doses causing asymptomatic and acute infections are being compared. We first have shown that even during lethal infection an infectious dose can play an important role.

We found that infection caused by administration of 10^3^ PFU of both TBEV strains to mice was characterized by two-wave viremia. The EK-328 strain demonstrated a significant increase in virus titers in blood of mice on the first day of infection. In the Absettarov-infected mice, the second peak of viremia with high virus titers in brain was significantly more pronounced than in mice infected with the EK-328 strain. Moreover, for the Absettarov strain, relatively high titers of neutralizing Abs coincided with the second peak of viremia, i.e., we observed a non-sterile immunity. TBE is characterized by non-sterile immunity when the infectious virus is isolated from the sera of patients wherein titers of neutralizing Abs are high (Levkovich et al., 1960). However, the mechanism of the non-sterile immunity is not well understood. Theoretically, several explanations can be proposed: the presence of escape mutants, induction of a high amount of low-avidity Abs, the difference in switching the synthesis from less specific IgM to more efficient IgG Abs, and an unknown ability of the virions to avoid interaction with Abs. Here we have demonstrated that virulent TBEV strains can differ by their ability to induce non-sterile immunity.

Both studied TBEV strains induced a surge of cytokine production during the terminal stage of infection, which could be considered as a cytokine storm. We could observe a significant increase in cytokine level in blood of the EK-328-infected mice only in the terminal stage of infection after injection of the high dose of the virus; while in the Absettarov-infected mice, the cytokine storm coincided with the appearance of the first disease symptoms. The cytokine storm in the CNS of infected mice at the terminal stage of infection was previously described after IC inoculation of TBEV (Salichev et al., 2013). In our experiments the differences between the studied TBEV strains might be associated with different speed of cytokines levels increase in the blood or brain as well as with a limited communication between blood and cerebrospinal fluid.

In the present study, highly virulent TBEV strains differed by characteristics of their interaction with DCs: level of virus reproduction, virus dose triggering IFN-α production, and impact on DCs' maturation. It is the first description of TBEV interaction with DCs, and the studied virulent TBEV strains differed significantly in the impact on DCs' maturation and Ag presentation to CD4^+^ lymphocytes.

Therefore, comparison of two TBEV strains similar by their virulence rate in mice after peripheral injection in equal viral dose showed significant variation in the process of infection and immune response activation:

(1) different dynamics of virus detection in blood of infected animals (Figure 1); (2) different ability to induce non-sterile immunity; (3) different dynamics of IFN-α, -γ, TNF-α, and IL-12 synthesis during the early stages of infection (before 28 h p.i.); (4) different dynamics of the synthesis of the cytokines at days 1 and 7 p.i.; (5) different dynamics of cytokine storm in blood of infected animals during the terminal stages of infection; and (6) different characteristics of virus interaction with DCs: level of reproduction, influence on DCs' maturation, and induction of IFNs.

We cannot associate the identified differences in the strain characteristics with the virus subtype. TBEV strains of various subtypes have demonstrated significant differences in their virulence in animal experiments (Il'enko et al., 1974; Kozlovskaya et al., 2010; Palus et al., 2013). In order to establish a correlation between the virus subtype and its pathogenetic and immunogenetic characteristics, extensive studies with the use of several strains belonging to different TBEV subtypes are required.

We realize that the present data could only partially be projected to the features of the TBEV population, which circulates in ticks in nature. The strains used in the present study underwent more than five passages through the mouse brains and as a result they could increase their virulence in laboratory animals. Nevertheless, two general conclusions can be made: (1) the virus dose during the lethal infection determines the characteristics of the immune response; (2) highly virulent TBEV strains can differ in the characteristics of their interaction with the host immune system. In this study we have tried to present more or less general picture of experimental TBE, defining the effects of the infectious dose and strain peculiarities. These data are important for understanding the immune response during TBE and can be utilized in development or testing of the novel compounds for TBE prophylaxis or treatment.

## Author contributions

AS and GK designed the experiments; AS, OM, VK, LG, YK, LK, YR, AL, and LR performed the experimental work; AS, OM, NA, LK, LR, and GK prepared the manuscript for publication; all authors agreed on publication of the manuscript.

## Funding

This work was supported by the Russian Science Foundation grant #15-14-00048. The funders had no role in study design, data collection and interpretation, or the decision to submit the work for publication.

### Conflict of interest statement

The authors declare that the research was conducted in the absence of any commercial or financial relationships that could be construed as a potential conflict of interest.
